# Respiratory health and inflammatory markers - Exposure to respirable dust and quartz and chemical binders in Swedish iron foundries

**DOI:** 10.1371/journal.pone.0224668

**Published:** 2019-11-01

**Authors:** Lena Andersson, Ing-Liss Bryngelsson, Alexander Hedbrant, Alexander Persson, Anders Johansson, Annette Ericsson, Ina Lindell, Leo Stockfelt, Eva Särndahl, Håkan Westberg

**Affiliations:** 1 Department of Occupational and Environmental Medicine, Faculty of Medicine and Health, Örebro University, Örebro, Sweden; 2 Department of Medical Sciences, School of Medicine and Health, Örebro University, Örebro, Sweden; 3 Inflammatory Response and Infection Susceptibility Centre (iRiSC), Örebro University, Örebro, Sweden; 4 Unit of Occupational and Environmental Medicine, Sahlgrenska Academy, University of Gothenburg and Sahlgrenska University Hospital, Gothenburg, Sweden; Telethon Institute for Child Health Research, AUSTRALIA

## Abstract

**Purpose:**

To study the relationship between respirable dust, quartz and chemical binders in Swedish iron foundries and respiratory symptoms, lung function (as forced expiratory volume FEV_1_ and vital capacity FVC), fraction of exhaled nitric oxide (FENO) and levels of club cell secretory protein 16 (CC16) and CRP.

**Methods:**

Personal sampling of respirable dust and quartz was performed for 85 subjects in three Swedish iron foundries. Full shift sampling and examination were performed on the second or third day of a working week after a work free weekend, with additional sampling on the fourth or fifth day. Logistic, linear and mixed model analyses were performed including, gender, age, smoking, infections, sampling day, body mass index (BMI) and chemical binders as covariates.

**Results:**

The adjusted average respirable quartz and dust concentrations were 0.038 and 0.66 mg/m^3^, respectively. Statistically significant increases in levels of CC16 were associated with exposure to chemical binders (p = 0.05; p = 0.01) in the regression analysis of quartz and respirable dust, respectively. Non-significant exposure-responses were identified for cumulative quartz and the symptoms asthma and breathlessness. For cumulative chemical years, non-significant exposure–response were observed for all but two symptoms. FENO also exhibited a non significant exposure-response for both quartz and respirable dust. No exposure-response was determined for FEV_1_ or FVC, CRP and respirable dust and quartz.

**Conclusions:**

Our findings suggest that early markers of pulmonary effect, such as increased levels of CC16 and FENO, are more strongly associated with chemical binder exposure than respirable quartz and dust in foundry environments.

## Introduction

Concerns about aerosol exposure in the iron foundry industry have largely been related to respirable quartz (CAS no14080-60-7), primarly because quartz exposure has been linked to increased risks of silicosis [1, 2], lung cancer [3, 4, 5] and cardiovascular disease and mortality [6, 7, 8].

Several studies have examined acute and chronic effects of aerosol exposure in foundry workers, focusing on respiratory symptoms and signs as well as lung function. A study in a small iron foundry in the Arabian Gulf States reported significant increases in the incidence of symptoms such as cough, phlegm and wheezing, non-significant increases in FVC and FEV_1_, and a significant change in the VC related to respirable dust exposures for exposed workers [9]. An Australian study investigated symptoms and lung function in a steel foundry, and no significant differences in chonic effects between the aftercast jobs and other job types were detected. However, high exposures to furfuryl alcohol and formaldehyde were determined in the general foundry areas [10]. A Finnish study in iron foundries related dusty jobs to an increased prevalence of chronic bronchitis, but not to any impairment of the lung function [11]. Respiratory impairment was investigated in a Brazilian study, in which duration of employment was used as a proxy for exposure. Significant excess of chronic bronchitis was related to duration of employment, but there was no detectable correlation between exposure and FEV_1_ or FVC [12]. Respiratory abnormalities (deviations from normal values) were investigated in a Taiwanese study [13], and a significantly increased risk for those employed > 10 years was noted. Respiratory symptoms and lung function were investigated in a Turkish study on iron foundries, in which the exposed group exhibited significantly higher incidence of symptoms compared to controls. The group exposed to very high respirable dust levels (10 mg/m^3^ respirable dust) exhibited no significant increase in FVC and FEV_1_ compared to the controls, but did exhibit a significant incidence of asthma [14]. In a British study, foundry workers were found to exhibit higher FEV_1_ and FVC, and a non-significantly higher prevalence of most respiratory symptoms, compared to controls [15]. An Iranian study on foundry core makers revealed statistically significant effects of high levels of silica, formaldehyde and trimethylamine exposure on FVC [16]. In a Swedish study, nitrogen wash out tests revealed impaired lung function among furan binder exposed workers indicating acute restrictiveness (not chronic) at high concentrations of furfuryl alcohol and formaldehyde [17].

In recent years, more sensitive indicators of inflammatory processes in the lung have been used to predict respiratory impairment or diseases. Nitric oxide (FENO), an indicator of primarily eosinophilic inflammation in the respiratory tract [18], produced in the bronchi or alveoli, was investigated in a Finnish study on foundry workers together with other inflammatory and coagulatory markers. A link between dust and quartz exposure was also investigated, revealing a positive association between cumulative dust exposure and FENO [19]. Serum levels of the surfactant protein, known as club cell secretory protein 16 (CC16), a marker of pulmonary inflammation or epithelial damage, has also been used to identify early signs of lung damage due to dust and chemical exposures [20].

We have previously presented exposure–response data for systemic inflammatory markers and exposure to quartz and respirable dust in iron foundries. Participants in the high-exposure groups with respect to some of the measured particle types exhibited significantly elevated levels of serum amyloid a (SAA), fibrinogen and the coagulation factor VIII [21]. Here, we investigate a possible exposure–response relationship between exposures to respirable dust, quartz, and chemical binders on one hand and respiratory symptoms, local and systemic biomarkers of airway effects and lung function on the other.

## Methods

### Study group

The study was performed at three Swedish iron foundries, one large and two smaller, situated in the middle of Sweden, employing 440, 90 and 25 workers, respectively. The number of day-shift workers at the foundries are 350, 80 and 25, respectively. These foundries produced some 100,000, 25,000 and 2,000 tons of castings per year, respectively. The castings produced are mainly based on iron and gray iron alloys and include products such as motor heads and blocks for trucks, large components for the wind power industry, and large/medium lego castings. The jobs and departments included in the study were sand preparation, core making, moulding, casting, shake out operations, fettling, inspection, and maintenance and repair. Melters, moulders and fettlers represented the majority of the jobs. The sand used for moulding was mostly green sand comprising silicates, quartz, carbon black, bentonite (clay and water) but chemical binders such as furan (furfuryl alcohol, urea, phenol, formaldehyde, p-toluenesulphonic acid or phosphoric acid) and silicate ester (sodium meta silicate and organic ester) were added to the silica sand, particularly in the smaller foundries. All mechanized and manual moulding and casting was performed at the foundries. The most common core binder was epoxy-SO_2_ and sodium silicate but coldbox (isocyanate-MDI, polyol, phenol formaldehyde) resin with an amine as catalytic agent, was also used as core binders in the foundries [22]. The study’s participants were foundry workers, predominantly from the day shift. In total, 80 men and 5 women participated in the study. Their mean age was 43 years, ranging from 21 to 67, evenly distributed when 10-year age classes were compared. They had worked in the same workplace for 12 years on average, ranging up to 45 years of employment. In total, 83 participants reported their smoking habits, 17 were current smokers, 18 were ex-smokers and 48 were never smokers.

Written informed consent was obtained for all individual participants included in the study. The study was approved by the Regional Ethical Review Board, Uppsala, dnr. 2015/066, including the informed consent procedures.

### Study design

The measurements were conducted between March 2015 and September 2016 during 6 separate four-days campaigns. Each campaign involved air sampling of respirable dust and quartz, biological sampling of blood, and investigation of lung function by spirometry and measuring FENO in exhaled air (S1 Fig). Air sampling of respirable dust and quartz was performed on the second or third working day after a work-free weekend. Blood sampling was performed on the same day as the air sampling, starting in the afternoon (3–4.30 p.m.), and was followed up after two days, i.e., on day 4 or 5 at work. Two blood samples were collected for each subject during each campaign. The post shift sampling design was initended to control for circadian variation. On the same day as the air sampling, the participants lung function was studied by spirometry and measuring FENO levels as a marker of inflammation. This was done before and after shifts and was followed up after two days, i.e., on day 4 or 5 at work. All participants completed a questionnaire, containing items relating to their current and previous working conditions, height and weight, smoking habits, medication, and symptoms of respiratory irritation, infection or other inflammation, which could influence the measured biomarker levels and respiratory outcomes. All participants were at work during our investigation. Seven workers reported suffering from chronic diseases such as thyroid imbalance, diabetes type 2, scoliosis, hypertension, depression, asthma or chronic obstructive pulmonary disease (COPD).

### Aerosol measurements and quartz analysis

The respiratory tract comprises three regions, the extrathoracic (which includes the nose and throat), tracheobronchial and alveolar regions. Aerosols reaching the alveolar region are sampled as respirable dust. The exposure data presented here are in principal based on exposure measurements of respirable dust and quartz, used in our exposure-response analysis. The measurements were performed on the second or third day after a work-free weekend, on the day the when first blood sample was taken for the individual in question. Personal sampling of respirable dust was performed by collecting 8-hour full shift samples according to standards for measurement of respirable dust using SKC HD aluminium cyclones for 25 mm filters, operating at an air flow of 2.5 L/min [23]. SKC AircheckXR5000, and GSA SG 5100 personal sampling pumps were used in the study. Respirable crystalline quartz was determined by X-ray diffraction [24].

Measuring rigs were used for stationary measurements (area measurements), in particular to determine background air concentrations. These measurements were peformed in the various departments where the workers were present during their shifts, and included sampling of inhalable dust as well as respirable dust and quartz using the same techniques as for the personal samples.

### Marker of inflammation, CC16, CRP

The blood samples were centrifuged and stored at -70 ^o^C at the biobank (Dnr 13OLL718-5). High-sensitivity CRP was determined at the Clinical Research Center, Örebro University Hospital, Sweden. CC16 in serum was analyzed using a commercial ELISA-kit from R&D Systems (Minneapolis, United States) in-house at the Department of Occupational and Environmental Medicine, Sahlgrenska University Hospital, Göteborg.

### Lung function and nitric oxide in exhaled air (FENO)

Lung function was assessed using spirare, a hand-held PC-based spirometry flow sensor with bi-directional ultrasound transit time analysis (Diagnostica, Oslo, Norway), according to guidelines from the American Thoracic Society [25]. The spirometry was performed on the same day as the personal air sampling, before and after shift, and was followed up after two days, i.e., on day 4 or 5 at work. The results were expressed in terms of the percentage of predicted forced vital capacity (FVC) and forced expiratory volume in one second (FEV_1_), determined using gender-specific Swedish reference data, adjusting for age and body weight [26, 27].

FENO, an indicator of primarily eosinophilic inflammation in the respiratory tract [18], was investigated using a NIOX Mino apparatus (Aerocrine AB, Solna, Sweden) with an exhaled flow rate of 50 ml/s during measurement [28]. The measurements were performed in connection to the spirometry. Information on factors (smoking, infections, food) that may affect the FENO concentrations was collected from each subject. The results were expressed as the fraction of exhaled FENO in parts per billion (ppb).

### Exposure measures

Exposure was primarly determined and presented as 8 hr-time weighted averages (TWA, mg/m^3^) of respirable dust and quartz based on exposure measurements relating to acute effects. The respirable fraction served as a proxy for other dust fractions, i.e., inhalable dust. In our stationary measurement inhalable and respirable dust correlated. The stationary measurements served as proxies for the additional exposure measures and were performed in each department, where individuals with the studied job titles worked. Participants working in high exposure jobs wore respirators (particularly shake-out and fettling operations). The personal measurements for the 28 subjects using respirators were adjusted to account for the use of respirator, reflecting their true exposures. To adjust for respirator use, exposure was assumed to be zero while wearing mask and to be equal to the corresponding background air concentrations for the rest of the shift, giving an 8 hr-time weighted average (TWA) exposure for the shift [21]. As a sensitivity analysis, we also performed our regression analysis assuming no protection effect of the respirators for both respirable quartz and dust.

Exposures were also determined and presented as cumulative (mg/m^3.^year) or mean (mg/m^3^) respirable quartz and dust exposure. We used data from a mixed model analysis of quartz air concentrations to calculate cumulative quartz exposure measures based on extracted measurement data from our iron foundry database [29] and data on dust and quartz from 2006 to 2016 for the three studied foundries to specifically model exposures at those three foundries. In this way, respirable quartz and dust exposures were determined (as concentrations) for different 10 year time periods in our three foundries, and for the job titles caster, core maker, fettler, furnace and ladle repair, maintenance, melter, moulder, sand mixer, shake out operator and transporter.

Exposures to chemical agents affecting the airways, i.e., the furan binder, were also accounted for in the analysis [17, 30]. The use of furan binder is known to generate emission of furfurylalcohol and formaldehyde in foundries. We used both cross-shift and cumulative exposure measures in this adjustment, chemical exposure (high exposure group; moulders, casters) (low exposure group: all other jobs) current exposure (8-hour TWA) to reflect current exposure and chemical exposure years (high exposure group: years of employment as a moulder or caster, (low exposure group: all other jobs) was used to reflect chronic effects and cumulative exposure.

### Statistical analysis

For descriptive purposes, the aerosol concentrations of respirable dust and quartz are presented by job title as 8-hour TWAs for a full workday (TWA). The reported results are adjusted for the use of respirators. Standard parameters such as the arithmetic mean (AM), standard deviation (SD), geometric mean (GM) geometric standard deviation (GSD) and range were calculated based on the log-normal distribution of all measurements (Tables 1 and 2).

**Table 1:** Exposure concentration levels of respirable quartz by job title, adjusted for the use of respirators.

Job title	N	AM	Median	SD	GM	GSD	Min	Max
Sand preparation	4	0.026	0.026	0.018	0.020	2.4	0.0064	0.046
Melting	13	0.027	0.0088	0.051	0.0093	4.5	0.0010	0.19
Core making	5	0.065	0.025	0.089	0.013	13	0.00090	0.21
Moulding	14	0.021	0.018	0.021	0.012	3.5	0.0010	0.077
Casting	4	0.0086	0.0090	0.0036	0.0080	1.6	0.0044	0.012
Shake out	5	0.080	0.077	0.055	0.067	1.9	0.035	0.17
Fettling and blasting	18	0.077	0.0044	0.19	0.0097	7.0	0.0010	0.61
Inspection	4	0.017	0.0098	0.022	0.0080	4.7	0.0019	0.048
Maintenance inlc cleaning	4	0.022	0.024	0.012	0.019	2.2	0.0059	0.035
Transport	7	0.020	0.017	0.014	0.015	2.3	0.0039	0.041
Others	7	0.0065	0.0058	0.0043	0.0051	2.3	0.0011	0.014
Total	85	0.038	0.014	0.094	0.012	4.4	0.00090	0.61

N: number of measurements, AM: arithmetic mean, SD: standard deviation, GM: geometric mean, GSD: geometric standard deviation

**Table 2:** Prevalence of mucous membrane and respiratory symptoms in foundry workers, percentage of answers (missing cases in parenthesis).

Symptom	N	Yes %	No %
Diagnosed asthma?	84 (1)	7.1	91.8
Asthma attack last 12 months?	84 (2)	1.2	97.6
During the last year. did you experience:			
Whistling or wheezing in your chest during the last 12 months?	83 (2)	11.8	85.9
Attacks of breathlessness during the last 12 months?	84 (1)	7.1	85.9
Dripping or blocked nose more than 1 month??	83 (2)	18.8	78.8
Dripping or blocked nose, itching or dripping nose the last 2 weeks?	85 (1)	41.2	57.6
Fever attacks related to work?	85 (2)	2.4	97.6
Attacks of cough without infection	85 (2)	18.8	78.8
Attacks of cough without infection at least 3 months	85 (20)	4.7	71.8
Attacks of cough with phlegm	83 (2)	25.9	71.8
Attacks of cough with phlegm at least 3 months	85 (20)	18.8	57.6
Attacks of cough with phlegm. at least 3 months, 2 years in a row	85 (23)	9.4	63.5

We also modelled respirable quartz and dust for the whole exposure period for all workers, basically by adding measurement data from 2005 to 2016 to our earlier mixed model analysis derived for some Swedish iron foundries, including those studied here [31]. The fixed effects were time period, type of foundry, and job title. The estimates from this model allowed us to identify factors affecting quartz concentration levels and to use these to calculate cumulative respirable quartz and dust exposures for each individual in our study. Adjustments to the measured exposure to chemical binders, i.e., furfuryl alcohol and formaldehyde were made on the basis of high and low exposure groups. These assessments compared high and low exposure groups using furfuryl alcohol and formaldehyde exposure measurements as proxies [17, 30].

Logistic regression analysis was used to evaluate exposure-responses for respirable quartz and dust, symptoms and signs. The determinants were age (>45; <45 years); gender (male, female); respirable dust (<0.1, 0.11–0.99, >1.0 mg/m^3^); respirable quartz (<0.01, 0.011–0.05; >0.05 mg/m^3^), cumulative respirable quartz (<0.1, 0.11–0.99; >1.0 mg/m^3^ years); cumulative respirable dust (<1.99, 2–9.99, >10 mg/m^3^ years); smoking habits (non-smoker, ex-smoker, smoker), chemical exposure (moulder, caster vs other jobs) based on current exposure (8-hour TWAs), and chemical exposure years (years as a moulder or caster vs. other jobs) based on chronic cumulative exposure. Separate analyses were performed for each measure of particle- and chemical binder exposure. Symptoms with less than 5 positive findings were omitted in the regression analysis. Multiple linear regression was used to study the chronic effects of cumulative exposure to respirable dust and quartz on FEV_1_, FVC, FENO, CC16 and CRP. The determinants for FEV_1_ and FVC were respirable dust (<1.99, 2–9.99, >10 mg/m^3^ years); respirable quartz (<0.1, 0.11–0.99; >1.0 mg/m^3^ years); smoking habits (non-smoker, ex-smoker, smoker); chemical exposure years (years as a moulder or caster vs other jobs) as a measure of chronic, cumulative exposures; and BMI (body mass index). For FENO and CC16 analysis, age (<45, >45 years), infection and gender (male, female) were also included as determinants.

A linear mixed model was used to describe the relationship between respirable quartz and dust exposure and FEV_1_, FVC, FENO, CRP and CC16 based on morning and afternoon sampling, with repeated afternoon sampling 2 days after the initial sample collection. Our design and the use of a mixed model made it possible to consider within and between worker variability based on our repeated measurements of CC16 and CRP. The exposure measurements were converted into 8-hour TWA levels for respirable dust (<0.1, 0.11–0.99, >1.0 mg/m^3^), respirable quartz (<0.01, 0.011–0.05; >0.05 mg/m^3^), cumulative respirable dust (<1.99, 2–9.99, >10 mg/m^3^ years), and cumulative respirable quartz (<0.1, 0.11–0.99; >1.0 mg/m^3^ years). In addition, sampling day (time), gender (male, female), BMI, smoking habits (never smoker, ex smoker, current smoker), age (< 45 years, >45 years), chemyears (cumulative exposure), chemexp (TWA), and infection were included. The output of the mixed model analysis is reported in terms of B values and 95% confidence intervals. To evaluate statistical significance, we used a significance threshold of p < 0.05.

All analyses were performed with SPSS version 22.0. Details of the regression and mixed model analysis are presented in S1 Appendix.

## Results

### Exposure

The mean 8-hour TWA for respirable quartz determined by personal sampling of the 85 participants was 0.052 mg/m^3^, with individual TWAs ranging from 0.0010 to 0.61 mg/m^3^. For respirable dust, the corresponding mean and range were 0.85 mg/m^3^ and 0.060 to 9.7 mg/m^3^ (not shown in table). For the personal measurements and for the 28 individuals using respirators, we calculated adjusted exposure concentrations based on non-adjusted, average, and background-adjusted respirable dust and respirable quartz concentrations. The adjusted average respirable quartz and dust concentrations were 0.038 and 0.66 mg/m^3^, respectively (Table 1). The highest adjusted quartz exposures were determined in the core making, shake out, and fettling departments, for which the mean adjusted exposures were 0.065, 0.080, and 0.077 mg/m^3^, respectively.

### Symptoms and lung function

Several mucous membrane and respiratory symptoms exhibited high (> 15%) prevalence among participants, including a dripping or blocked nose for > 1 month (18.8%), dripping or blocked nose in the last two weeks (41.2%), coughing fits with no associated infection (18.8%), coughing fits with phlegm (25.9%), and blocked nose for > 1 month (18.8%) (Table 2).

A logistic regression analysis revealed no significant exposure-response between symptoms and cumulative respirable quartz exposure adjusted for age, smoking, gender and chemical years (Table 3). However, non-significant exposure-responses were identified for most symptoms when comparing the high and low exposure groups.

**Table 3:** Symptoms and exposure to cumulative respirable quartz (mg/m^3^ year) and chemical binders (chem years)—logistic regression analysis presented as odds ratios adjusted for age, smoking and gender.

Exposure	Symptom								
	Asthma			Wheezing			Breathlessness		
Resp quartz mg/m^3^ year	OR	p	95% CI	OR	p	95% CI	OR	p	95% CI
<0.1	***1***	0.17		1	0.99		***1***	0.90	
0.11–0.99	***3*.*10***	0.36	0.26–36.10	0.91	0.90	0.18–4.48	***1*.*19***	0.88	0.12–11.46
1.0+	***27*.*40***	0.06	0.86–87.30	0.95	0.96	0.12–7.68	***1*.*99***	0.66	0.10–41.82
Chem years	***1*.*04***	0.56	0.92–1.17	***1*.*02***	0.68	0.93–1.12	***1*.*04***	0.51	0.92–1.18
	**Nose dripping >1 month**			**Nose dripping last 2 week**			**Cough without infection**		
Resp quartz mg/m^3^ year	OR	p	95% CI	OR	p	95% CI	OR	p	95% CI
<0.1	1	0.88		1	0.33		1	0.47	
0.11–0.99	1.39	0.61	0.38–5.11	0.51	0.23	0.17–1.53	0.50	0.33	0.13–1.99
1.0+	0	0.99	0	0.32	0.20	0.05–1.85	0.27	0.29	0.02–3.06
Chem years	***1*.*00***	0.99	0.88–1.14	***1*.*09***	0.08	0.99–1.21	***1*.*03***	0.50	0.95–1.12
	**Cough phlegm**			**Cough with phlegm 3 months**					
Resp quartz mg/m^3^ year	OR	p	95% CI	OR	p	95% CI			
<0.1	1	0.74		1	0.50				
0.11–0.99	0.62	0.44	0.18–2.09	0.80	0.77	0.18–3.60			
1.0+	0.69	0.67	0.12–3.99	0.32	0.25	0.05–2.22			
Chem years	***1*.*03***	0.38	0.96–1.11	0.93	0.20	0.84–1.04			

OR: odds ratios, p: p-value, 95% CI: 95% confidence interval, bold italic numbers: non-significant exposure-response

A corresponding analysis of symptoms, cumulative respirable dust and chemical years exposure revealed a similar pattern, although a statistically significant increase in the odds ratio for chemical years and nose dripping was determined (OR 1.12; p = 0.05 (Table 4). Additionally, a non-significant exposure response was determined for both respirable dust and quartz exposure with respect to asthma and breathlessness. No significant exposure response was determined for any symptom with respect to daily TWA exposure to quartz and chemical exposure (measured over complete shifts, data not in table). However, non-significant increases in the incidence of wheezing (OR 2.28), nose problems (OR 2.89), and cough (OR 3.38) were determined for chemical exposure. The corresponding analysis of respirable dust and chemical exposures revealed a non-significant exposure response for nose irritation for respirable dust (ORs 1, 1.02 and 2.93, respectively). Wheezing (OR 1.73), nose irritation (OR 3.17) and cough showed the same pattern for exposure to chemical binders.

**Table 4:** Symptoms and exposure to cumulative respirable dust exposures (mg/m^3^ year) and chemical binders (chem years)—logistic regression analysis presented as odds ratios adjusted for age, smoking and gender.

Exposure	Symptom								
	Asthma			Wheezing			Breathlessness		
Resp dust mg/m^3^ year	OR	p	95% CI	OR	p	95% CI	OR	p	95% CI
<1.99	***1***	0.13		1	0.87		1	0.43	
2–9.99	***1*.*78***	0.66	0.13–24.09	1.03	0.98	0.20–5.30	0.23	0.28	0.02–3.40
>10	***13*.*29***	0.07	0.78–227.34	0.64	0.67	0.08–4.98	1.16	0.91	0.10–14.01
Chem years	***1*.*03***	0.59	0.92–1.17	***1*.*02***	0.64	0.93–1.13	***1*.*05***	0.43	0.93–1.20
	**Nose dripping >1 month**			**Nose dripping last 2 week**			**Cough without infection**		
Resp dust mg/m^3^ year	OR	p	95% CI	OR	p	95% CI	OR	p	95% CI
<1.99	1	0.75		1	0.08		1	0.11	
2–9.99	1.68	0.45	0.44	0.29	0.05	0.09–0.97	0.18	0.05	0.03–0.96
>10	0	0.99	0	0.25	0.07	0.05–1.14	0.73	0.71	0.13–4.00
Chem years	***1*.*02***	0.78	0.82–1.18	**1.12**	**0.05**	**1.00–1.25**	***1*.*04***	0.33	0.96–1.13
	**Cough phlegm**			**Cough with phlegm 3 months**					
Resp dust mg/m^3^ year	OR	p	95% CI	OR	p	95% CI			
<1.99	1	0.25		1	0.89				
2–9.99	0.40	0.17	0.10–1.47	1.42	0.65	0.30–6.67			
>10	0.28	0.14	0.05–1.53	1.41	0.70	0.25–8.03			
Chem years	***1*.*05***	0.23	0.97–1.13	0.93	0.17	0.83–1.03			

OR: odds ratios, p: p-value, 95% CI: 95% confidence interval, bold numbers: statistical significant OR, bold italic numbers: non-significant exposure-response

The average lung function measurements—vital capacity (FVC) and expiratory volume (FEV_1_)—for the three different samples were low, ranging from 86.9 to 89.6% of expected levels in the Swedish population (Table 5). Additionally, the variation within and between individuals was high: 53.9 to 124.0% of the expected value for FVC, and 56.1 to 127.5% for FEV_1_. Notably, FEV_1_ and FVC declined over the course of a shift, by 0.5 and 0.6%, respectively.

**Table 5:** Lung function FVC, FEV_1_ as percentage of expected values, FENO and CC16.

	N	AM	Median	Min	Max	SD
**FVC %** Pre shift (1) sampling day 1	85	87.5	87.7	53.9	119.4	12.5
Post shift (2) sampling day 1	84	86.9	86.9	55.6	119.9	12.4
Post shift (3) sampling day 3	83	87.8	87.9	56.1	124.0	12.4
Δ FVC (post shift-pre shift)	84	-0.5	0.0	-18.8	8.2	4.2
Δ FVC post shift (day 3-day 1)	82	1.0	0.7	-18.7	18.4	4.7
**FEV**_**1**_**%**						
Pre shift (1) sampling day 1	85	89.6	90.5	57.7	127.5	13.5
Post shift (2) sampling day 1	84	88.9	88.5	59.6	127.5	13.0
Post shift (3) sampling day 3	83	87.8	87.9	56.1	124.0	12.4
Δ FEV_1_ (post shift-pre shift)	84	-0.6	-0.4	-20.4	10.2	4.5
Δ FEV_1_ post shift (day 3-day 1)	82	0.8	0.6	-11.3	18.4	4.5
**NO ppb**						
Pre shift (1) sampling day 1	85	16.5	14.0	5.0	82.0	12.0
Post shift (2) sampling day 1	85	16.0	12.0	5.0	62.0	10.0
Post shift (3) sampling day 3	83	16.7	14.0	5.0	64.0	11.0
Δ NO (post shift-pre shift)	85	-0.5	0.0	-20.0	10.0	4.3
Δ NO post shift (day 3-day 1)	83	0.8	1.0	-11.0	24.0	4.9
**CC16 μg/L**						
Post shift (2) sampling day 1	85	22.4	20.7	9.2	50.2	9.1
Post shift (3) sampling day 3	83	22.8	20.4	8.8	53.3	9.7
Δ CC16 post shift (day 3-day 1)	83	0.5	0.3	-5.3	6.0	2.2

N: number of measurements, AM: arithmetic mean, SD: standard deviation

No statistically significant exposure response was observed for a chronic effect of FEV_1_ and FVC related to cumulative respirable quartz and dust exposure. In fact, lung function increased slightly with exposure (Table 6). For FENO, the average levels were low (16 to 16.7 ppb), although some measurements were above 50 ppb. Linear regression models revealed non-significant increases in FENO levels related to respirable quartz and dust exposure; these increases were as high as 6.28 ppb per >1.0 mg/m^3^ year respirable quartz and 4.91 per >10 mg/m^3^ year respirable dust (Table 6).

**Table 6:** FEV_1_ and FVC (% of expected), FENO, CC16 and CRP morning day 1, cumulative exposure to respirable quartz, respirable dust (mg/m^3^ year) and chemical binders (chem years)–multiple linear regression adjusted for smoking, BMI, age and gender.

Exposure	FEV_1_%			FVC %					
Resp quartz mg/m^3^ year	B	p	95% CI	B	p	95% CI			
<0.1	0			0					
0.11–0.99	1.72	0.60	-4.75–8.18	1.07	0.72	-4.92–7.06			
>1.0	-0.69	0.89	-10.16–8.78	0.03	0.99	-8.76–8.81			
Chem years	-0.21	0.33	-0.63–0.22	-0.10	0.63	-0.49–0.30			
	**FENO ppb**			**CC16 μg/L**			**CRP mg/l**		
Resp quartz mg/m^3^ year	B	p	95% CI	B	p	95% CI	B	p	95% CI
<0.1	***0***			0			0		
0.11–0.99	***1*.*70***	0.54	-3.77–7.80	-1.05	0.63	-5.36–3.26	-0.66	0.29	-1.90–0.57
>1.0	***6*.*28***	0.13	-1.97–14.50	***5*.*62***	0.09	-0.88–12.11	0.11	0.90	-1.75–1.98
Chem years	***0*.*17***	0.37	-0.21–0.55	**0.39**	**0.01**	**0.10–0.69**	-0.19	0.66	-0.11–0.07
	**FEV**_**1**_**%**			**FVC %**					
Resp dust mg/m^3^ year	B	p	95% CI	B	p	95% CI			
<1.99	0			0					
2–9.99	4.57	0.17	-2.05–11.18	4.66	0.13	-1.45–10.77			
>10	3.89	0.36	-4.43–12.20	2.69	0.49	-4.99–10.37			
Chem years	-0.26	0.22	-0.68–0.16	-0.15	0.44	-0.54–0.24			
	**FENO ppb**			**CC16 μg/L**			**CRP mg/l**		
Resp dust mg/m^3^ year	B	p	95% CI	B	p	95% CI	B	p	95% CI
<1.99	***0***			0			0		
2–9.99	***2*.*73***	0.35	-3.08–8.54	3.35	0.16	-1.30–7.99	-0.79	0.24	-2.11–0.54
>10	***4*.*91***	0.20	-2.62–12.44	2.82	0.35	-3.20–8.84	-0.40	0.63	-2.04–1.25
Chem years	***0*.*18***	0.36	-0.21–0.58	**0.31**	**0.05**	**0.00–0.63**	0.01	0.82	-0.07–0.09

B: regression coefficient, p: p-value, 95% CI: 95% confidence interval, bold numbers: statistical significant OR, bold italic numbers: non-significant exposure-response

### CC16, CRP

A comparison of high and low exposure groups revealed statistically significant exposure responses linking elevated CC16 levels to long-term exposure (expressed in terms of chemical exposure years) to chemical binders (i.e. furfuryl alcohol and formaldehyde). The B-values for this relationship determined by treating respirable dust and quartz exposure as fixed effects were B = 0.39 (p = 0.01) and B = 0.31 (p = 0.053), respectively (Table 6). These B-values indicate a 30 to 40% increase in the contribution of chemical binders to the model. Upon segmenting binder-exposed group based on duration of exposure (1–5, 6–10 and 10+ years), it was found that the group with the longest exposure duration exhibited significantly elevated mean (26 vs. 22 **μ**g/L) and median (23 vs 21 **μ**/L) CC16 levels. This trend was also observed in the corresponding linear regression analysis using cumulative quartz and chemical binder exposure measurements (B = 4.0 vs 0, non-significant, data not in table). Our sensitivity analysis regarding acute effects, assuming no effect of respirators, showed no difference in the logistic regression analysis, i.e., the effect of respirable quarts and dust on symptoms and irritative effects were the similar to our analysis with adjusted exposures. When analyzing the CC16 levels for the afternoon samples collected on days 1 and 3 using a mixed model analysis, a statistically significant exposure-response was identified upon comparing the high and low exposure groups (groups defined based on long-term exposure in chemical years; β = 0.408; p = 0.012, data not in table). The corresponding analysis of chemical exposure (cross-shift exposure) revealed no significant exposure-response. These data suggest an exposure-response for chemical binders based on cumulative exposure years. No exposure–response was determined for CRP, a marker of systemic inflammation, when analyzed for cumulative respirable quartz, respirable dust and chemical years (Table 6). Similar results was found when current exposure to respirable quartz, respirable dust and chemical exposure (TWA) were evaluated (data not in table).

## Discussion

### Main findings

The main finding of our study is an association between CC16 levels and long-term chemical exposure (expressed in chemical years) to binders, i.e., furfuryl alcohol and formaldehyde. Additionally, FENO levels increased over full shifts and exhibited non-significant exposure responses to quartz and respirable dust. Non-significant positive exposure responses to cumulative respirable quartz exposure were determined for the symptoms asthma and breathlessness, and to chemical years (cumulative exposures) for all but two symptoms. Non-significant exposure responses to daily quartz and chemical exposure (based on daily TWAs) were determined for the symptoms wheezing, nose problems and cough.

To our knowledge, this is one of the first reported study to examine the effects of respirable quartz, respirable dust and chemical agent exposure on respiratory symptoms and effects, markers of early inflammatory processes in the lung (FENO and CC16) in foundry environments. A key strength of this study is the determination of multiple quantitative exposure measures, including average daily levels and cumulative exposure measures for respirable quartz and dust as well as qualitative exposure to chemical binders (i.e., furfuryl alcohol and formaldehyde). We were also able to incorporate exposure data from 2005 to 2015 for the foundries examined in this work, thereby completing our original database [29]. Although our personal aerosol sampling include only respirable fractions active in the small airways, a number of particle mass based measures were sampled (data not presented). Additional stationary sampling showed good statistically significant correlation between inhalable and respirable dust (r = 0.872, p<0.01), implying the use of the respirable fractions as a marker of inhalable dust. The blood sampling for CC16 was designed to avoid the influence of diurnal variation; sampling was performed at the end of a shift to facilitate comparisons based on temporal trends and other determinants [32]. In the design phase of the study, we excluded the use of a totally non-exposed reference group because the use of such a group could introduce socioeconomic bias. Instead, we relied on between-group (exposure classes) comparisons using exposure-response analyses for respirable quartz and dust. We also treated exposure to chemical binders (i.e., furfuryl alcohol and formaldehyde) as a binary variable (high/low exposed). In our exposure measurements, moulders and casters were treated as the high exposure group and were compared to foundry employees with other jobs. No measurements of furfuryl alcohol or formaldehyde were performed in our study; the exposure assessment was based on historical exposure measurements and the concept of similar exposure groups [17, 31, 33]. For the studied responses (lung function, symptoms, CC16, CRP and FENO), current exposure responses were analyzed over full shifts and long-term effects were analyzed based on morning samples and full shift exposure, together with cumulative exposures from our company-specific measurement database.

Particle sampling and analysis and blood sampling were performed using standard methods at accredited laboratories. The overall respirable quartz concentration measurements and those for specific job titles at the three foundries are consistent with previous reports [34].

Some 30% of the participating workers used respirators during some parts of their shifts (determined individually for each worker). For these workers, we calculated individual exposure measures based on the amount of time spent wearing a respirator; their exposure was assumed to be zero during this time, and to be equal to the background exposure for the relevant department during the remainder of their shift [21]. We consider this correction strategy to be the most reliable of those available.

Our study design is cross-sectional with respect to the medical investigations, but many of the respiratory symptoms and signs as well as lung function could be due to long term exposure. Our exposure data allowed us to determine average intensities (cross-shift exposure measures) as well as cumulative exposure measures expressed as mg/m^3^ years. In most cross–sectional studies, the exposure is only measured cross-shift and long–term exposure only estimated by duration of exposures (year). However, all workers entering the iron foundry environment passes mandatory health examinations when entering work as well as every third year for the high exposed workers, focusing on lung diseases related to quartz exposure. The test includes x-ray, questionnaires and lung function controls, revealing lung diseases, and preventing workers with disease to enter the iron foundry environment. There is a risk that our results may be biased by the healthy worker effect [35], i.e. that we may have analyzed only very healthy individuals. However, our spirometric lung function assessments did not reveal any extremely healthy workers; mean preshift FVC and FEV_1_ measurements ranged from 87.5 to 89.6% of the expected values [27]. Our finding would then represent exposure to quartz for workers passing health examinations and not the general population, implying considerations when a study like ours will be used for setting occupational exposure limits. We did not collect data on atopy, but consider that it is unlikely that atopic status is correlated with the exposure-response analyses in this study.

### CC16

Club cell protein 16 is a small protein secreted by bronchiolar club cells into the epithelial lining fluid of the lungs and is believed to protect against inflammation and oxidative stress. A small fraction normally passes through lung epithelial barrier into serum, where increased levels can be used as an early marker of lung injury [36]. In addition to increased leakage, serum CC16 levels may increase due to elevated expression in the respiratory tract during inflammation or decreased renal clearance [37]. Serum CC16 levels generally fall after chronic particle exposure (such as that resulting from tobacco smoking) due to the destruction of club cells; they generally rise after current exposure and in lung disorders that increase airway permeability [37, 38]. Studies have examined the response of CC16 serum levels to various exposures and in diverse populations, including garbage and waste water aerosols [39], asphalt workers [20] grain dust workers [40], wood smoke [41, 42], smoke [43, 44], ozone [45], chlorine [46] and outdoor air pollution particles [47]. In most cases, serum CC16 levels increased upon exposure to particles or chemical agents. However, decreased levels were observed among ski waxers after exposure [48] sewage workers [49] and diesel exhaust exposed workers [50], and offshore oil drill workers [51]. We observed no short term differences in serum CC16 between post-shift samples collected on day 1 and day 3, so our results provide no evidence that occupational exposure has short-term effects. The half life of CC16 in serum is short (hours), however, so it is possible that our sampling at the end of the shift missed transient effects of exposure in the earlier part of the shifts. Additionally, there was no association with long-term exposure to respirable quartz or dust. Long-term exposure to chemical binders (furfuryl alcohol and formaldehyde) was, somewhat surprisingly, associated with increased levels of serum CC16. This may be because the binder exposure levels observed in this work did not affect airways strongly enough to cause club cell destruction. The increase in CC16 levels could be due to the chronic airway effects of chemical binder exposure, which may increase epithelial permeability and induce CC16 upregulation as an anti-inflammatory response to airway irritants. Our findings were supported by an additional exposure response analysis that revealed a non-significantly increased inflammatory response among highly exposed groups. To our knowledge, no data on the relationship between CC16 levels and foundry work or chemical binder exposure have been presented previously. However, as described above, increased CC16 levels due to acute and chronic exposure have been reported in other occupationally exposed groups [39, 40, 49].

### CRP

CRP was analyzed as a marker of systemic inflammation to contrast our marker of local airway inflammation, CC16. We could not detect any exposure-response for CRP when respirable quartz, respirable dust and chemical binders was evaluated in the regression or mixed model analysis. This results were in line with findings in our previuos study on systemic inflammation in iron foundry workers [21]. However, a study in Finnish foundries analysed pulmonary inflammation using exhaled breath condensate, determining FENO and CRP (CC16 excluded). They found significant differences for NO and CRP levels when the high or low exposure groups of respirable quartz and dust were compared to controls, the CRP increased from 0.77 to 0.92 and 1.06 mg/L, respectively [19]. Our CRP mean concentration was 1.8 mg/L. The exposure-reponse in the Finnish study was based on cumulative exposures for respirable quartz and dust presented as unexposed, low (<2.5 mg/m^3^ years) and high (>2.5 mg/m^3^ years) exposure groups. The cumulative high exposure group in the Finnish study is twice as high as ours, which might explain the differences in CRP response between our studies. A Chinese occupational cohort of diesel exhaust exposed workers and an unexposed control group, presented decreased CC16 and increased CRP (CRP for exposed vs controls 0.91 vs 0.47 mg/L) [50]. FEV_1_ was also significantly increased in the control group. Furthermore, a Norwegian study on sewage plant and sewer net workers showed increased levels of CRP (ref 1.4, sew plant 1.9, sew net 3 mg/L) when compared to a control group [49]. In contrast, studies on exposure to grain dust [40] and tunnel construction workers [52] could not present any statistically significant effect on CRP.

### Symptoms

#### Asthma

The foundry environment and the use of furan binders in particular were listed as irritants causing asthma and/or COPD in a recent review [53]. While this statement was based solely on a case report, impaired lung function has also been reported for foundry workers using furan binders [17]. Our data suggest a non-significant exposure response-relationship for exposure to chemical binders, respirable quartz and diagnosed asthma. For asthma, very high odds ratios for the high exposure group was determined.

#### Chronic bronchitis

In a Finnish study, iron foundry workers were separated into high (> 0.2 mg/m^3^), intermediate (0.1 mg/m^3^), and low (<0.1 mg/m^3^) exposure groups based on respirable quartz levels. In this population, 7% reported cough, 5% phlegm, and 2% chronic bronchitis. No exposure response was determined [11]. In a Taiwanese study of foundry workers, the incidences of chronic cough, chronic phlegm, and bronchitis ranged from 5 to 8%, 10 to 17%, and 2 to 10%, respectively, at average respirable dust concentrations of 1.9 to 2.8 mg/m^3^ for highly exposed workers [13]. No statistically significant differences were observed when comparing the exposed to a control group of administrative personnel. A Brazilian publication focusing predominantly on iron foundries reported an overall chronic bronchitis prevalence of 10% with a significant increase in incidence among workers with a job duration of 10–16 years; job years was used as a proxy for exposure. No exposure response was established [12]. A Canadian study on iron foundry workers (using railway workers as a control group) detected an elevated prevalence of cough, phlegm and chronic bronchitis, although none were statistically significant. The mean respirable dust exposure among the foundry workers was 1.26 mg/m^3^ [15]. A similar study from the Arabian Gulf States reported prevalences of cough and phlegm of 38 and 25%, respectively, at very high respirable dust concentrations: the average levels for the low, medium, and high exposure groups were 0.26, 5.31 and 21 mg/m^3^, respectively [9].

Among the never smokers participating in our study, the prevalences of chronic cough, chronic phlegm, and bronchitis were 15%, 25%, and 22%, respectively. We found no exposure response between chronic bronchitis and exposure to quartz (based on a cumulative respirable quartz level of 1.0 mg/m^3^ years, corresponding to an exposure of 0.1 mg/m^3^ for 10 years). The other studies discussed above reported much higher respirable quartz and dust concentrations but did not report any exposure-response analysis. However, in our study we found increased odds ratios for most symptoms when high and low culumative exposure to chemical binders was analysed, suggesting respiratory symptoms related rather to chemical binder exposure than to respirabel dust and quartz.

### Lung function, dust and quartz

In the Finnish study cited above [11], dust exposure itself did not affect FEV_1_ or FVC; if anything, both quantities were higher at high dust concentrations. Selective turnover of workers from dusty to less dusty jobs was proposed as an explanation for this observation. In the Taiwanese study, the FEV_1_ and FVC values for workers exposed to rather high average respirable dust concentrations ranging from 1.9 to 2.8 mg/m^3^ did not differ significantly from those for a control group [13].

A Brazilian study used duration of exposure as a proxy when evaluating FEV_1_ and VC by logistic regression analysis. No significant exposure response was detected; in this case, the duration of exposure for the high exposure group was > 16 years [12].

In the Canadian iron and steel foundry, exposed workers exhibited statistically significant changes when comparing to the control group. Using duration of exposure as a proxy, however, no exposure response was determined at mean respirable dust concentrations of 1.26 mg/m^3^ [15]. In the study on foundry workers in the Arabian Gulf states, a comparison of the exposed and control groups revealed significant changes for FEV_1_ but not for FVC. Job type significantly affected FVC but not FEV_1_, and multivariate analysis indicated an inversely significant exposure response, i.e., lung function improved (in terms of FVC) as exposure increased. However, there was no detectable exposure response in terms of FEV_1_ when participants were divided into dust exposure classes, even though the average respirable dust exposure of the high exposure group was 21 mg/m^3^ [9]. The only study presenting cross-shift changes of FEV_1_ and FVC showed decreased FEV_1_ (6.4%) and FVC (4.0%) cross-shift at very high respirable quartz air concentrations, on average 0.25 mg/m^3^ [9]. The smaller decreases in our study, 0.5 and 0.6% respectively, could be explained by lower respirable quartz levels, on average 0.038 mg/m^3^.

Our findings are consistent with those of studies examining participants exposed to much higher respirable dust and quartz levels than the workers at the studied foundries. Notably, this is even the case for the (non-significant) inverse exposure response for respirable quartz and dust that was detected in the analysis of the quantitative data. The absence of an effect on lung function among our high cumulative exposure groups (inclusion criteria: 1 mg /m^3^ year for respirable quartz, corresponding to 0.1 mg/m^3^ exposure over 10 years, and 10 mg/m^3^ year for respirable dust, corresponding to 1 mg/m^3^ exposure for 10 years) is reasonable given the observation of similar absences for groups exposed to much higher levels of respirable quartz and dust.

### Furan binder(chemical years and chemical exposure)

Non-significant reductions in FEV_1_ (B = -0.21; p = 0.33) and FVC (B = -0.26; p = 0.22) were observed in relation to chemical years when analyzing the fixed effect of respirable quartz exposure, and a similar pattern was observed for respirable dust. This suggests an effect of furfuryl alcohol and formaldehyde for highly exposed workers (moulders, casters) that is stronger than that experienced by shake out and fettling workers. In a Swedish study [17], the TWA values for furfuryl alcohol ranged from 4 to 15 mg/m^3^, with short-term exposure levels reaching 100 mg/m^3^. In the same work, respirable dust concentrations ranged from 0.5 to 2 mg/m^3^, and respirable quartz from 0.01 to 0.11 mg/m^3^. The exposed group exhibited significant declines in FVC and FEV_1_ (i.e., a negative exposure response) over a full shift when compared to controls. However, analysis of morning spirometry data revealed no evidence of any effect of chronic exposure. In our study, both high and low exposure groups exhibited non-significant declines in both FEV_1_ and FVC relating to chemical exposure years (i.e., cumulative exposure to chemical binders and thus to furfuryl alcohol and formaldehyde).

### FENO

A Finnish study (earlier discussed regarding CRP levels) on pulmonary inflammation among foundry workers presented cumulative exposures to total dust and respirable dust and an exposure response analysis based on high and low dust exposure groups (>53 mg/m^3^ year and <53 mg/m^3^ year, respectively) and high and low cumulative quartz exposure groups (>2.5 mg/m^3^ year and < 2.5 mg/m^3^ year, respectively) [19]. No statistically significant exposure-response was determined, but a trend was noted when the high and low exposed were compared to the controls. The high or low exposed compared to the unexposed groups differed significantly in FeNO levels, however no exposure response could be determined [19]. These data are consistent with our observation of a non-significant positive response to respirable quartz and dust exposure for both FEV_1_ and FVC.

To summarize the published data on respiratory symptoms, lung function and respirable quartz and dust, several studies have reported differences between exposed and control groups, but many of the respiratory symptoms as well as changes in lung function could be due to much higher levels of respirable quartz and dust than the foundry employees studied in this work were exposed to. The cumulative respirable quartz exposure for the highly exposed group in our analysis was > 1 mg/m^3^ year, corresponding to 0.1 mg/m^3^ for 10 years, while the cumulative respirable dust exposure for this group was 10 mg/m^3^ year, corresponding to 1 mg/m^3^ for 10 years. Hence, there is a risk that our results indicate that the exposure levels and durations of exposure to quartz and respiratory dust among the studied foundry workers are too low to appreciably impair respiratory health.

## Conclusions

We measured air exposure levels of respirable quartz and dust among workers at Swedish iron foundries and qualitatively assessed their exposure to chemical binders, i.e., furfuryl alcohol and formaldehyde. The adjusted average respirable quartz and dust concentrations were 0.038 and 0.66 mg/m^3^, respectively. Statistically significant increased levels of CC16 were detected for high cumulative exposures to chemical binders compared to low exposed. Non-significant exposure-responses were detected for asthma, breathlessness and FENO for several exposure measures. Our findings suggest that changes in early effect markers such as CC16 and FENO are associated more strongly with exposure to chemical binders than to respirable quartz and dust exposure.

## Supporting information

S1 Fig Flow chart of the procedure of the study for each participant. (PDF)

S1 File Questionnaire air pollution and health. (PDF)

S2 File Questionnaire nitric oxide in exhaled air (FENO). (PDF)

S1 Appendix Statistical analysis. (PDF)
