# Supplementary figures and images for: Respiratory health and inflammatory markers - Exposure to respirable dust and quartz and chemical binders in Swedish iron foundries
Source: PLoS One. 2019 Nov 1;14(11):e0224668. doi: 10.1371/journal.pone.0224668 (PMC6824619; doi:10.1371/journal.pone.0224668)

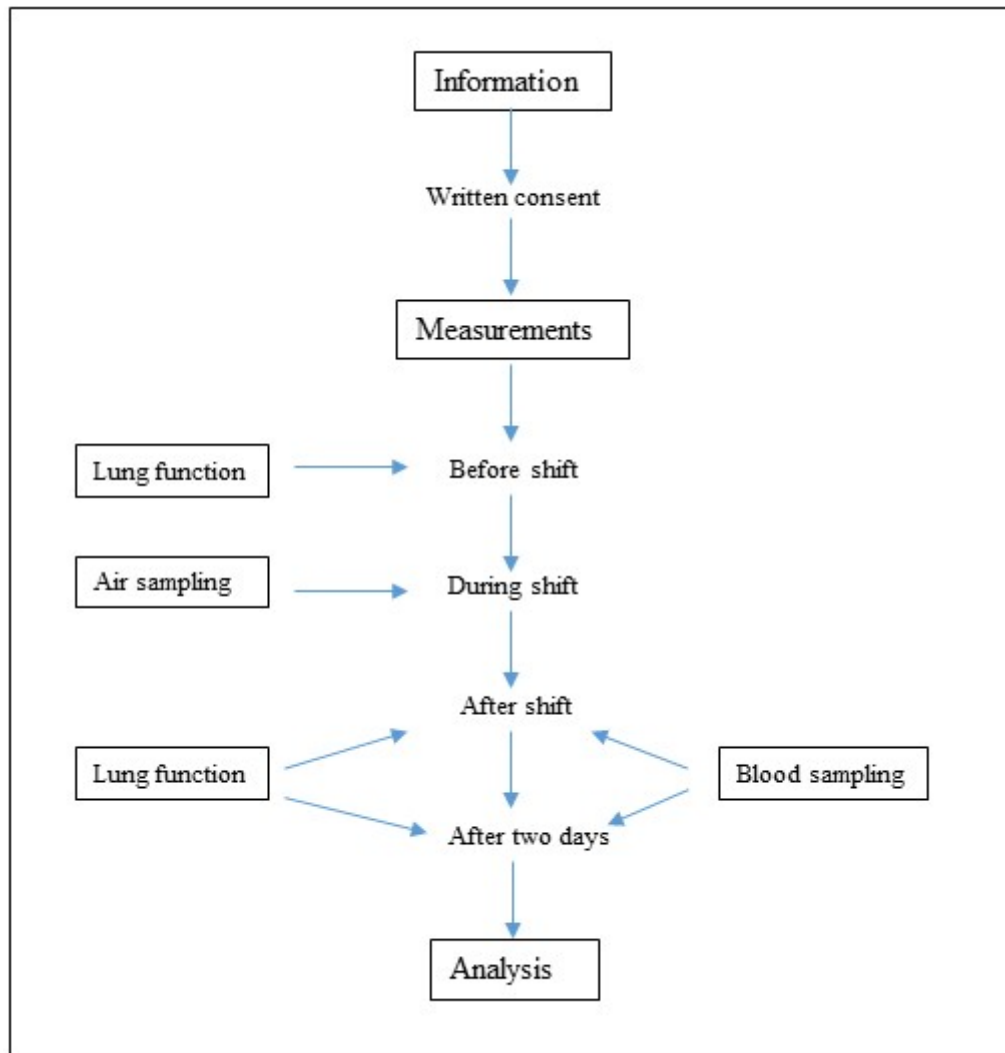

**Fig 1. Flow chart of the procedure of the study for each participant.**

Supplement: S1 Fig — (PDF) [file pone.0224668.s001.pdf]
