# Supplementary material for: Respiratory health and inflammatory markers - Exposure to respirable dust and quartz and chemical binders in Swedish iron foundries
Source: PLoS One. 2019 Nov 1;14(11):e0224668. doi: 10.1371/journal.pone.0224668 (PMC6824619; doi:10.1371/journal.pone.0224668)
Supplement: S1 File — (PDF) [file pone.0224668.s002.pdf]

Questionnaire
Air pollution and health

The questionnaire contains questions about your work, leisure and health status. Your answers are important for us to be able to draw the correct conclusions about possible health impact of air pollution at ysour workplace.

The purpose of the project is to describe the exposure to airborne particles in foundries through blood and air sampling.

Try to answer all questions as completely as possible.

The answers are sent to us in the enclosed reply envelope - (or, easiest, to leave the envelope at the time of sampling).

The questionnaire is kept with the same confidentiality as a medical record. The results will be reported as group values so that no individual can be recognized.

Contact any of us if you have any questions.

Anette Ericsson
Occupational health nurse
Dep. of Occupational and Environmental Medicine
Örebro University Hospital
Phone 019-602 24 95
E-mail annette.ericsson@regionorebrolan.se

Lena Andersson
Occupational hygienist
Dep. of Occupational and Environmental Medicine
Örebro University Hospital
Phone 019-602 35 92
E-mail lena.andersson4@regionorebrolan.se

Anahita Keloushani
MD
Dep. of Occupational and Environmental Medicine
Örebro University Hospital
Phone 019-602 24 43
E-mail anahita.keloushani@regionorebrolan.se

Date ……………….

Code ……………….

CURRENT WORK

Work place ……………………………………………………………………………………………..
Profession ……………………………………………………………………………………………..
Current working tasks ………………………………………………………………………………

Been working with current working tasks	since year	…………….
Been working at current work place	since year	…………….
Average working hours, including overtime	hours/week	…………….

	Yes	No
Do you work or have you worked shifts?	☐	☐
If yes, between which years?	…………..	…………….
	…………..	…………….

How often do yopu use hearing protection at work?	☐Always	☐Most of the day	☐Sometimes	☐Not at all

How much of the day do you spend in a noisy environment?	Hours/ working hours	………..	(for example 2/8)

TOBACCO HABITS

Are you

☐Never smoker	☐Former smoker<br>What year did you quit? ……………	☐Current smoker<br>Average number of cigarettes/day<br>Pipe, cigar ……………
☐Never snuffer	☐Former snuffer<br>What year did you quit? ……………	☐Current snuffer<br>Average number of snuff cans/week ………

AIR POLLUTIONS IN THE ENVIRONMENT

Enter environmental factors that you regurarly (not temporary) are exposed to:	Exposed to in current work environment?		Exposed to in leisure time?	
	Yes	No	Yes	No
Dust (stone, wood, textile)	☐	☐	☐	☐
Tobacco smoke (passive smoking)	☐	☐	☐	☐
Smoke (fume, soldering)	☐	☐	☐	☐
Exhaust gases	☐	☐	☐	☐
Gas and solvents (ammonia, thinner, tri)	☐	☐	☐	☐
Other air pollutants, which:	☐	☐	☐	☐

………………………………………………………………………..

HAVE YOU DONE ANY OF THE FOLLOWING DURING THE LAST 2 WEEKS?

	No	Yes, the last 2 weeks	Yes, the last 2 days
Worked with house/holiday home	☐	☐	☐
- Sanded and painted	☐	☐	☐
- Carpentry	☐	☐	☐
- Isolated	☐	☐	☐
- Annat	☐	☐	☐
Worked with agriculture	☐	☐	☐
- Handeled hay or straw (harvested, stored)	☐	☐	☐
- Caring animal (cow, horse, pig or similar)	☐	☐	☐
- Other	☐	☐	☐
Sanded and/or welded on car or similar	☐	☐	☐
Holiday in a big city	☐	☐	☐
- Extra employment	☐	☐	☐
- With what? …………………………………….			
- Barbecued	☐	☐	☐
Used anti-inflammatory drugs	☐	☐	☐

If yes, which drugs?

....

……………………………………………………….

……………………………………………………….

SYMTHOMS FROM THE AIR WAYS

	Yes	No
Have you been diagnosed with asthma by a doctor?	☐	☐
If yes, what year ……………………….		
Do you currently use any medication (spray, inhalation powder or tablets for asthma)?	☐	☐
Have you had any asthma attack during the last 12 months?	☐	☐
Have you had any whistling or wheezing in your chest during the last 12 months?	☐	☐
Have you during the last 12 months had any attack of breathlessness?	☐	☐
Have you had or do you have any nose symthoms (blocked nose, dripping nose) for more than 1 month?	☐	☐
If yes, what year did these symthoms start ……………………….		
Have you had blocked nose, itchy or dripping nose and/or been sneezing the last 2 weeks?	☐	☐
Have you had attacs of fever or chills, that seems to be connected to your work and not from a normal cold or flu?	☐	☐
Do you attacks of cough (cough without phlegm)?	☐	☐
If yes, have you had attacks of cough for, in total, 3 months during a year?	☐	☐
Do you have attacks of cough with phlegm?	☐	☐
If yes, do you have attacks of cough with phlegm for at least 3 months during the last year?	☐	☐
If yes, did you have attacks of cough with phlegm for at least 3 months, 2 years in a row?	☐	☐
If yes, what year did these symthoms start? …………		

INFECTIONS

	Yes	No
Have you had any infection the last 2 weeks?	☐	☐
Have you had any injury (cuts, bone fracture, wound infection) the last 2 weeks?	☐	☐

THANK YOU FOR YOUR PARTICIPATION!

Frågeformulär
Luftföroreningar och hälsa

Frågeformuläret innehåller frågor om Ditt arbete, fritid och hälsotillstånd.

Dina svar är viktiga för att vi ska kunna dra rätt slutsatser om ev. hälsopåverkan av luftföroreningarna på Din arbetsplats.

Syftet med projektet är att genom blod- och luftprovtagning beskriva exponeringen för luftburna partiklar i gjuter.

Försök att besvara alla frågor så fullständigt som möjligt.

Svaren skickas till oss i medföljande svarskuvert -

(eller, enklast, att lämna kuvertet vid provtagningstillfället).

Frågeformuläret förvaras med samma sekretess som en medicinsk journal. Resultaten kommer att redovisas som gruppvärden så, att ingen enskild person kan kännas igen.

Kontakta någon av oss om du har några frågor.

Anette Ericsson
Företagssköterska
Arbets- och miljömedicin
Universitetssjukhuset Örebro
Telefon 019-602 24 95
e-post annette.ericsson@regionorebrolan.se

Lena Andersson
Yrkeshygieniker
Arbets och miljömedicin
Universitetssjukhuset Örebro
Telefon 019-602 35 92
e-post lena.andersson4@regionorebrolan.se

Anahita Keloushani
Specialistläkare
Arbets- och miljömedicin
Universitetssjukhuset Örebro
Telefon 019-602 24 43
e-post anahita.keloushani@regionorebrolan.se

Datum ……………….

Kod ……………….

NUVARANDE ARBETE

Arbetsplats ………………………………………………………………………………………..
Yrke ………………………………………………………………………………………..
Nuvarande arbetsuppgifter …………………………………………………………………………….

Arbetat med nuvarande arbetsuppgifter sedan år …………….
Arbetat på nuvarande arbetsplats sedan år …………….
Arbetstid i genomsnitt, inklusive övertid tim/vecka …………….

	Ja	Nej
Arbetar du eller har du arbetat skift?	☐	☐
Om ja, mellan vilka år	…………..	…………….
	…………..	…………….

Hur ofta använder du hörselskydd när du är ute i processen? ☐ Alltid ☐ Oftast ☐ Ibland ☐ Inte alls

Hur stor del av dagen vistas du i bullrig miljö Tim/arbtid ………. (t ex 2/8

TOBAKSVANOR

Är du

☐ Aldrig rökare	☐ Före detta rökare	☐ Rökare<br>Röker i genomsnitt cigaretter/dag
	Vilket år slutade du? ……………	Pipa, cigarrr, cigarill ……………
☐ Aldrig snusare	☐ Före detta snusare	☐ Snusare
	Vilket år slutade du? ……………	Snusar i genomsnitt dosor/vecka ………

LUFTFÖRORENINGAR I MILJÖN

Ange miljöfaktorer som du regelbundet (ej tillfälligt) är utsatt för:	Utsatt för i nuvarande arbetsmiljö?		Utsatt för på fritiden?	
	Ja	Nej	Ja	Nej
Damm (sten, trä, textil m m)	☐	☐	☐	☐
Tobaksrök (passiv rökning)	☐	☐	☐	☐
Rök (svetsrök, lödrök m m)	☐	☐	☐	☐
Motoravgaser	☐	☐	☐	☐
Gas och lösningsmedel (ammoniak, thinner, tri m m)	☐	☐	☐	☐
Andra luftföroreningar, vilka:	☐	☐	☐	☐

………………………………………………………………………..

HAR DU UNDER DE SENASTE 2 VECKORNA GJORT NÅGOT AV FÖLJANDE?

	Nej	Ja, senaste 2 veckorna	Ja, senaste 2 dagarna
Arbetat med hus/fritidshus	☐	☐	☐
- Slipat och målat	☐	☐	☐
- Snickrat	☐	☐	☐
- Isolerat	☐	☐	☐
- Annat	☐	☐	☐
Arbetat på lantbruk	☐	☐	☐
- Hanterat hö eller halm (skördat, lagrat m m)	☐	☐	☐
- Skött djur (ko, häst, svin eller liknande)	☐	☐	☐
- Annat	☐	☐	☐
Slipat och/eller svetsat på bil eller liknande	☐	☐	☐
Semestrat i storstad	☐	☐	☐
- Arbetat extra	☐	☐	☐
- Med vad? …………………………………….			
- Grillat mat sista dagarna	☐	☐	☐
Använt anti-inflammatoriska läkemedel	☐	☐	☐

Om ja, vilka läkemedel?

....

……………………………………………………….

……………………………………………………….

BESVÄR FRÅN UFTVÄGARNA

	Ja	Nej
Har du fått diagnosen astma av läkare?	☐	☐
Om JA,vilket år ……………………….		
Använder du för närvarande någon medicin (spray, inhalationspulver eller tabletter mot astma)?	☐	☐
Har du haft något astmaanfall under de senaste 12 månaderna?	☐	☐
Har du haft pip eller väsningar i bröstet de senaste 12 månaderna?	☐	☐
Har du någon gång de senaste 12 månaderna haft någon attack av andnöd?	☐	☐
Har du eller har du haft näsbesvär (nästäppa, rinnsnuva mm) i mer än 1 månad?	☐	☐
Om JA, vilket år startade dessa besvär ……………………….		
Har du haft nästäppa, kliande eller rinnande näsa och/eller nysningar senaste 2 veckorna?	☐	☐
Har du haft attacker av feber eller frossa, som verkar ha samband med ditt arbete och som inte förefaller bero på vanlig förkylning eller influensa?	☐	☐
Brukar du ha torrhosta (hosta utan upphostning)?	☐	☐
Om JA, har Du torrhosta minst 3 månader sammanlagt om året?	☐	☐
Brukar du hosta upp slem (upphostning)?	☐	☐
Om JA, hostar du upp slem under minst 3 månader sammanlagt på året?	☐	☐
Om JA, har du gjort detta under minst 3 månader minst 2 år i rad?	☐	☐
Om JA, vilket år startade dessa besvär? …………		

INFEKTIONER

	Ja	Nej
Har du haft någon infektion senaste 2 veckorna?	☐	☐
Har du haft någon skada (t ex skärsår, benbrott, sårinfektion) senaste 2 veckorna?	☐	☐

TACK FÖR DIN MEDVERKAN!
