# Supplementary material for: Respiratory health and inflammatory markers - Exposure to respirable dust and quartz and chemical binders in Swedish iron foundries
Source: PLoS One. 2019 Nov 1;14(11):e0224668. doi: 10.1371/journal.pone.0224668 (PMC6824619; doi:10.1371/journal.pone.0224668)
Supplement: S2 File — (PDF) [file pone.0224668.s003.pdf]

Nitric oxide in exhaled air (FENO)

Code: ____________________________

	Date			
	Time<br>**Before shift**		Time<br>**After skift**	
	Yes	No	Yes	No
1. Do you use<br>a. asthma spray with cortisone?	☐	☐		
b. angina medication?	☐	☐		
If yes, which medication do you use?	____________________			
2. Have you had a cold the last 3 weeks?	☐	☐		
3. Do you have a cold now?	☐	☐	☐	☐
4. Do you have a fever?	☐	☐	☐	☐
5. Have you used nose spray the last 24 hours?	☐	☐	☐	☐
If yes, how many hours ago?	____________________			
6. Do you have runny nose?	☐	☐	☐	☐
blocked nose?	☐	☐	☐	☐
itchy nose?	☐	☐	☐	☐
sneezing?	☐	☐	☐	☐
7. Do you smoke?	☐	☐		
If yes, how many hours ago?	____________________			
8. Does anybody else in your home smoke?	☐	☐		
9. Do you snuff?	☐	☐		
If yes, how many hours ago did you snuff?	____________________			
10. Have you had salad, spinach, beet, fruit, more than 4 potatoes or charcuteri the last 4 hours?	☐	☐	☐	☐
11. Have you had coffe or light beer the last 4 hours?	☐	☐	☐	☐
12. Have you been physically straining for the last hour?	☐	☐	☐	☐
13. How did you go to work?	____________________			

FENO measurement time before shift_____ ppb

time after shift_____ ppb

Kvävemonoxid i utandningsluft

Kod: ______________________________

	Datum			
	Tid<br>**Före skift**		Tid<br>**Efter skift**	
	Ja	Nej	Ja	Nej
1. Använder du				
a. astmaspray med kortison?	☐	☐		
b. kärlkrampsmediciner?	☐	☐		
Om ja, vilket preparat använder du?	______________			
2. Har du varit förkyld de senaste 3 veckorna?	☐	☐		
3. Är du förkyld nu?	☐	☐	☐	☐
4. Har du feber?	☐	☐	☐	☐
5. Har du använt nässpray senaste dygnet?	☐	☐	☐	☐
Om ja, för hur många timmar sedan?	______________			
6. Har du snuva?	☐	☐	☐	☐
nästäppa?	☐	☐	☐	☐
klåda i näsan?	☐	☐	☐	☐
nysningar?	☐	☐	☐	☐
7. Röker du?	☐	☐		
Om ja, för hur många timmar sedan?	______________			
8. Röker någon annan i hemmet?	☐	☐		
9. Snusar du?	☐	☐		
Om ja, för hur många timmar sedan snusade du?	______________			
10. Har du ätit sallad, spenat, rödbetor, frukt, mer än 4 potatisar eller charkuterivaror de senaste 4 timmarna?	☐	☐	☐	☐
11. Har du druckit kaffe eller lättöl de senaste 4 timmarna?	☐	☐	☐	☐
12. Har du ansträngt dig fysiskt senaste timmen?	☐	☐	☐	☐
13. Hur har du tagit dig till arbetet?	______________			

NO-mätning kl före skift_____ ppb kl efter skift_____ ppb
