# Supplementary material for: Respiratory health and inflammatory markers - Exposure to respirable dust and quartz and chemical binders in Swedish iron foundries
Source: PLoS One. 2019 Nov 1;14(11):e0224668. doi: 10.1371/journal.pone.0224668 (PMC6824619; doi:10.1371/journal.pone.0224668)
Supplement: S1 Appendix — (PDF) [file pone.0224668.s004.pdf]

Appendix. Statistical analysis

For descriptive purposes, the aerosol concentrations of respirable dust and quartz are presented by job title as 8-hour TWAs for the full workday. Data is presented as adjusted for the use of respirators. Standard parameters such as arithmetic mean (AM), standard deviation (SD), geometric mean (GM) geometric standard deviation (GSD) and range were calculated for the log normal distribution of all the measurements (Table 1).

Logistic regression analysis was applied to evaluate exposure-response for respirable quartz and dust, symptoms and signs. The regression presents the log of the odds of having a symptom or not as an additive function of a + bx. This equation can be generalized to include any number of explanatory variables:

$$\text{Log}\ [p/(1-p)] = a + b_1x_1 + b_2x_2 + b_3x_3 + ... + b_nx_n.$$

The determinants were age b_1 (>45; <45 years); gender b_2 (male, female); respirable dust b_3 (<0.1, 0.11-0.99, >1.0 mg/m^3); respirable quartz b_3'(<0.01, 0.011-0.05; >0.05 mg/m^3), smoking habits b_4 (non-smoker, ex- smoker, smoker) and *chemical exposure* b_4 (moulder, caster vs other jobs) reflecting acute exposure (8-hr TWA); *chemical exposure years* b_4' (moulder, caster years vs other year in other jobs) reflecting chronic, cumulative exposures. Separate analyses was performed for each measure of exposure. Symptoms with less than 5 positive findings were omitted in the regression analysis.

Multiple linear regression was applied to study chronic effect by cumulative exposures of respirable dust and quartz on FEV_1, FVC, FENO and CC16

$$y = a + b_1x_1 + b_2x_2 + b_3x_3 + ... + b_nx_n.$$

The determinants for FEV_1 and FVC were respirable dust b_1 (<1.99, 2-9.99, >10 mg/m^3 years); respirable quartz b_1' (<0.1, 0.11-0.99, >1.0 mg/m^3 years); smoking habits b_2 (non-

smoker, ex- smoker, smoker), *chemical exposure years b_3 (*moulder, caster years vs other jobs) reflecting chronic, cumulative exposures; BMI b_4 (body mass index). For FENO and CC16 analysis, age b_5 (<45, >45 years) and gender b_6 (men, women) were added to the analysis as determinants.

A linear mixed model was applied to describe the relation between respirable quartz and dust and FEV_1, FVC, FENO and CC16, morning sampling 1 and afternoon sampling 2 (over shift) as well as afternoon sampling of FEV_1 and FVC sample 2 and 3 (2 days between) sampling. Our design and the use of mixed model offered a possibility to consider within- and between worker variability for our repeated measurements. Exposure (respirable dust b_1 (<1.99, 2-9.99, >10 mg/m_3 years); respirable quartz b_1´ (<0.1, 0.11-0.99; >1.0 mg/m^3 years), sampling day (time) (x_2), gender (x_3) (men, women), BMI (x_4), smoking habits (x_5) (non-smoker, ex-smoker, smoker), age (< 45 years, >45 years (x_6), *chem years* (x_7)´ (cumulative exposure), *chem exp* (x_7) (TWA) and infection (x_8) were included.

The estimates (β) of the fixed effects from the model allowed us to identify factors affecting inflammatory markers. The mixed model applied has the following equation:

$$y = \mu + \beta_1 x_1 + \beta_2 x_2 + \beta_3 x_3 + \beta_4 x_4 + \beta_5 x_5 + \beta_6 x_6 + \beta_7 x_7 + \beta_8 x_8 + \varepsilon$$

The result of the mixed model analysis is presented as odds ratios and 95 % confidence interval. For statistical significance we used $p<0.05$. The analyses were performed with the SPSS software 22.0.
